# Malnutrition in Alzheimer’s Disease, Dementia with Lewy Bodies, and Frontotemporal Lobar Degeneration: Comparison Using Serum Albumin, Total Protein, and Hemoglobin Level

**DOI:** 10.1371/journal.pone.0157053

**Published:** 2016-06-23

**Authors:** Asuka Koyama, Mamoru Hashimoto, Hibiki Tanaka, Noboru Fujise, Masateru Matsushita, Yusuke Miyagawa, Yutaka Hatada, Ryuji Fukuhara, Noriko Hasegawa, Shuji Todani, Kengo Matsukuma, Michiyo Kawano, Manabu Ikeda

**Affiliations:** 1 Department of Neuropsychiatry, Faculty of Life Sciences, Kumamoto University, Kumamoto, Japan; 2 Faculty of Human Life Science, Shokei University, Kumamoto, Japan; University of Nottingham, UNITED KINGDOM

## Abstract

Malnutrition among dementia patients is an important issue. However, the biochemical markers of malnutrition have not been well studied in this population. The purpose of this study was to compare biochemical blood markers among patients with Alzheimer’s disease (AD), dementia with Lewy bodies (DLB), and frontotemporal lobar degeneration (FTLD). A total of 339 dementia outpatients and their family caregivers participated in this study. Low serum albumin was 7.2 times more prevalent among patients with DLB and 10.1 times more prevalent among those with FTLD than among those with AD, with adjustment for age. Low hemoglobin was 9.1 times more common in female DLB patients than in female AD patients, with adjustment for age. The levels of biochemical markers were not significantly correlated with cognitive function. Family caregivers of patients with low total protein, low albumin, or low hemoglobin were asked if the patients had loss of weight or appetite; 96.4% reported no loss of weight or appetite. In conclusion, nutritional status was worse in patients with DLB and FTLD than in those with AD. A multidimensional approach, including blood testing, is needed to assess malnutrition in patients with dementia.

## Introduction

Cognitive function and nutrition are thought to have strong correlations. In general, weight loss associated with malnutrition often precedes the onset of dementia and then increases in pace with progression of the disease [1]. Longitudinal cohort studies have reported that malnutrition itself is a risk factor for cognitive decline [2]. A possible correlation has also been found between deficiency in specific nutrients (e.g., vitamin B12 and folate) and decreased cognitive function [3,4].

People with cognitive decline have various eating and swallowing problems. Eating and swallowing problems, along with the behavioral and psychological symptoms of dementia, can strongly affect nutritional status. Kai et al. reported that more than 80% of patients with Alzheimer’s disease (AD) have eating and swallowing disturbances [5]. Other forms of dementia are also associated with several types of eating problems. In frontotemporal lobar degeneration (FTLD), changes in eating behavior (e.g., changes in appetite, food preferences, eating habits, and other oral behaviors) are frequently seen [6]. In dementia with Lewy bodies (DLB), eating and swallowing problems result from various factors, including Parkinsonism [7]. The reported prevalence of swallowing problems among dementia patients is 13%–57% [8].

Eating and swallowing problems increase the risk of malnutrition. Droogsma et al. reported that about 14% of community-dwelling people with dementia were at risk of malnutrition [9]. Among the dementia diagnostic groups, DLB patients have an especially high reported rate of malnutrition [10]. These previous studies assessed malnutrition with the Mini Nutritional Assessment [11], which consists of six aspects: decreased food intake, weight loss, mobility, psychological stress or acute disease, presence of dementia or depression, and Body Mass Index. This tool is convenient and reliable, but it lacks information about biochemical marker levels. To our knowledge, no studies have used biological blood markers to examine nutritional status in different types of dementia.

It is important to detect malnutrition in dementia patients as early as possible. In the usual clinical setting, some clinicians may judge patients’ nutritional status only by asking family caregivers about appetite and weight change in patients. However, it is unknown whether information provided by family caregivers about nutritional status agrees with biochemical marker levels.

The purpose of this study was i) to compare biochemical blood marker levels in three types of neurodegenerative dementia: AD, DLB, and FTLD; and ii) to examine the relationship between patient malnutrition and caregiver awareness of the patient’s appetite and weight change.

## Methods

### Participants

This study was approved by the Human Ethics Review Committee of Kumamoto University. After a complete description of all study procedures was provided, written informed consent was obtained from patients and their family caregivers.

Consecutive outpatients at the Dementia Clinic of the Department of Neuropsychiatry, Kumamoto University Hospital and their family caregivers participated in this study. Inclusion criteria were 1) diagnosis of AD, DLB, or FTLD; 2) living at home; 3) age under 90 years; 4) no physical illness which would affect biochemical blood values, such as hepatic or kidney dysfunction or severe inflammation. First-visit patients with AD were recruited from April 2010 to November 2014, and those with DLB and FTLD from June2007 to November 2014. This difference of the survey period is due to the difference of prevalence of each diagnosis, that is, DLB and FTLD is rare diagnoses compared with AD.

Patients who fulfilled the above criteria (n = 339) were examined by senior neuropsychiatrists who have adequate experience with dementia patients. All patients underwent routine laboratory tests, neuroimaging studies such as magnetic resonance imaging and single-photon emission computed tomography, and standard neuropsychological examinations. Dementia was diagnosed according to the Diagnostic and Statistical Manual of Mental Disorders, 3rd edition, revised [12]. Patients were diagnosed with probable AD, defined according to the National Institute for Neurological and Communicative Disorders and Stroke and the Alzheimer’s Disease and Related Disorders Association [13]; probable DLB, defined according to the Consensus Criteria for the clinical diagnosis of DLB, 2005 [14]; or probable FTLD, defined according to the Consensus Criteria for the clinical diagnosis of FTLD [15].

### Measures

Total protein, albumin, and hemoglobin were assessed as biological blood markers related to malnutrition. Malnutrition was defined as total protein level <6.5 g/dL and serum albumin <3.5 g/dL [16, 17]. For hemoglobin, we set abnormal levels according to sex (<12 g/dL for men; <11 g/dL for women) because men and women have different hemoglobin levels [18].

Other variables assessed were age, sex, years of education, duration of illness, cognitive function, and appetite/weight change. Cognitive function was assessed with the Mini-Mental State Examination (MMSE) [19]. The MMSE is a widely used cognitive screening test that assesses the severity of cognitive impairment. Scores range from 0 to 30, with higher scores indicating better cognitive functioning. To assess changes in appetite and weight, we used a part of the Japanese version of the 12-item Neuropsychiatric Inventory [20, 21]. The Neuropsychiatric Inventory, a semi-structured interview with a caregiver, is used to assess the behavioral and psychological symptoms of dementia. We used a subquestion in the domain of “Appetite and eating change.” Patients with “loss of appetite” or “weight loss” during the past month we regarded as “having loss of appetite or weight.”

### Statistical analysis

First, we compared the prevalence of low total protein, serum albumin, and hemoglobin among AD, DLB, and FTLD groups with the chi-square test and Z-test for comparison of column proportions. Next, we conducted polytomous logistic regression analysis to clarify the likelihood of low total protein, serum albumin, and hemoglobin among the three groups after adjusting for age. Then, the Pearson’s correlation coefficients were calculated between biochemical markers and demographic and clinical factors. Finally, we examined the perception of patients’ nutritional status among caregivers of patients with biochemical indications of malnutrition.

All tests were two-tailed and the significance levels were set at p<0.05. All statistical analyses were performed with SPSS 23.0J for Windows (IBM SPSS Japan, Tokyo, Japan).

## Results

Demographic and clinical characteristics of the patients are shown in Table 1. Mean patient age was lower in the FTLD group than in the AD and DLB groups. There were no significant differences among the three diagnostic groups in sex, years of education, duration of illness, or MMSE scores.

**Table 1:** Demographic and other characteristics of the three diagnostic groups.

	AD (n = 238)	DLB (n = 65)	FTLD (n = 36)	*F/ χ*^*2*^	p
Mean age, years^a^	77.3	78.4	68.1	23.5	<0.001
Sex, M/F (n)	69/169	26/39	12/24	2.923	0.232
Mean years of education	10.7	10.2	10.9	1.465	0.233
Mean duration of illness, years	2.5	2.4	2.8	0.623	0.537
Mean MMSE score	19.3	18.6	17.7	1.441	0.238

AD, Alzheimer’s disease; DLB, Dementia with Lewy bodies; FTLD, Frontotemporal lobar degeneration; MMSE, Mini-Mental State Examination

^a^ AD, DLB > FTLD (p<0.001)

There were significant differences in total protein among the three diagnostic groups, but in post hoc testing, the differences were not significant. The prevalence of low albumin was significantly higher among patients with DLB (6.2%) than among those with AD (0.8%) (p<0.05). Among female patients, the prevalence of low hemoglobin was significantly higher in the DLB group (10.3%) than in the AD group (1.2%) (p<0.05). There were no significant differences in hemoglobin levels among male patients (Table 2).

**Table 2:** Prevalence of abnormal blood marker values (%).

	AD (n = 238)	DLB (n = 65)	FTLD (n = 36)	*χ*^*2*^	p	
Total protein <6.5 g/dL	3.4	9.2	11.1	6.194	0.045	
Albumin <3.5 g/dL	0.8	6.2	5.6	8.041	0.018	AD<DLB
Hemoglobin <12 g/dL (males)	5.8	7.7	0	0.931	0.628	
Hemoglobin <11 g/dL (females)	1.2	10.3	4.2	0.035	0.011	AD<DLB

Chi-square test and Z-test were used to compare column proportions.

Polytomous logistic regression analysis adjusting for age among the three diagnostic groups showed that DLB and FTLD patients were 7.2 times and 10.1 times more likely, respectively, to have low serum albumin than AD patients (DLB: Wald = 4.983, p = 0.026; FTLD: Wald = 4.014, p = 0.045). Female DLB patients were also 9.1 times more likely to have low hemoglobin (Wald = 6.208, p = 0.013) than female AD patients. There were no significant differences among the three diagnostic groups in total protein or in hemoglobin among the men (Table 3).

**Table 3:** Odds ratio of abnormal levels of biochemical blood markers (reference = AD).

		Odds ratio (95% CI)	
	AD (n = 238)	DLB (n = 65)	FTLD (n = 36)
Total protein <6.5 g/dL	1	2.8 (0.9–8.5)	3.2 (0.7–14.0)
Albumin <3.5 g/dL	1	7.2 (1.3–40.4)*	10.1 (1.1–97.7)*
Hemoglobin <12 g/dL (male)	1	1.3 (0.2–7.5)	-
Hemoglobin <11 g/dL (female)	1	9.1 (1.6–52.1)*	6.3 (0.5–77.5)

*p<0.05

We calculated the Pearson’s correlation coefficient between each biochemical marker and demographic and clinical factors. Age was significantly correlated with albumin level in AD (*r* = −0.343, p<0.01) (Bonferroni-corrected p-value). There were no significant correlations between biochemical markers and other factors (duration of illness, years of education, and MMSE).

We examined the association between biochemical levels and caregivers’ perceptions of patients’ loss of appetite or weight. Of the 28 patients who were diagnosed with malnutrition based on total protein, serum albumin, or hemoglobin level, all but one family caregiver (96.4%) answered that the patient had neither loss of appetite nor weight loss.

## Discussion

This study is the first to clarify malnutrition according to type of dementia by assessing biochemical blood markers of nutritional status. In our study, the biochemical indicators of malnutrition were at lower levels in DLB and FTLD patients than in AD patients. In one of the few studies that have compared nutritional status by type of dementia, Roque et al. reported that malnutrition was more frequently seen in patients with DLB [10], which is consistent with our results. The prevalence of malnutrition among patients with FTLD was not higher than among those with AD and DLB in simple comparison. However, after adjusting for age among types of dementia, the prevalence of malnutrition was higher in the FTLD group than in the AD group. It would be generally natural to think that malnutrition in FTLD is rare, because eating problems such as preference for certain foods and overeating are frequently seen in FTLD. In fact, a previous study reported higher Body Mass Index in FTLD than in other types of dementia [22], although that study did not adjust for age among dementia diagnostic groups. However, because the onset of FTLD is generally earlier than that of other types of dementia and because nutritional status worsens with age, comparison with adjustment for age is more appropriate. FTLD patients have many types of eating problems and often want to eat only certain foods [6], which might lead to unbalanced dietary intake and malnutrition [23].

Older people are generally more likely to develop malnutrition as they age, so our finding that albumin level correlated with patient age in AD was reasonable. Meanwhile, biochemical levels were not correlated with cognitive function, which was inconsistent with a previous study [24]. On the other hand, it is reported that loss of appetite is seen regardless of the stage of dementia [5]. This difference could result in part from selection bias: previous studies have evaluated patients with more severe dementia living in hospitals or nursing homes, whereas our data are from patients with milder dementia. Our result suggests that malnutrition can occur at any stage of dementia among dementia outpatients.

Most of the family caregivers of dementia patients with malnutrition reported that their patients did not have loss of appetite or weight. This result suggests that interviews with family caregivers might not an accurate method of assessing nutritional status. A previous study that found that the nutritional status of dementia patients was associated with their caregivers’ nutritional status [25], suggesting that family caregivers of patients with malnutrition might not be aware of the patients’ nutritional status. Therefore, to assess the nutritional status of dementia patients, it is necessary not only to interview family caregivers but also to use objective indicators such as biochemical markers or body weight.

Although this is the first study to use biochemical blood markers to compare nutritional status among different types of dementia, there are some limitations to be addressed. The first and most important is that there is controversy regarding the validity of using biomarkers to assess nutritional status. To examine nutritional status precisely, it would be important to assess more various and broader aspects of nutritional state (e.g., other biochemical markers such as folate, vitamin B and D, BMI, meal surveys). Nonetheless, we think it is worth measuring core nutritional biomarkers, because some studies clarified that biochemical marker (total protein, albumin, and hemoglobin) and the Mini Nutritional Assessment was relatively correlated [24, 26]. The finding that feeding intervention for dementia patients improved albumin and hemoglobin levels [27] also supports the validity of assessing biochemical markers. Next, the sample size in FTLD and DLB is not enough because these are considerably rare diseases compared with AD [28]. A larger sample size would yield more reliable results. Third, it is also impossible to know whether differences in nutritional status among dementia diagnostic groups results from eating behavior that are typical for each diagnostic group, or if malnutrition itself causes certain types of dementia. The fourth limitation is that the participants were all outpatients and therefore their dementia was relatively mild and their level of nutrition was generally good. Our research should be viewed as preliminary for these important limitations, and further researches are needed that examine the possibility of malnutrition multidimensionally in DLB and FTLD using larger sample sizes.

In conclusion, serum albumin and hemoglobin were lower among patients with DLB and FTLD than among those with AD when adjusting for age. This is a preliminary study; more substantive research is needed to clarify the factors that cause differences in albumin and hemoglobin levels among dementia diagnostic groups. Also, family caregivers of dementia patients with biochemical markers of malnutrition did not recognize patients’ loss of appetite or weight loss. It is important to understand the discrepancy between biomarkers and caregivers’ impression of nutritional status in the clinical setting. Multidimensional nutritional assessment in patients with dementia (e.g., BMI, meal survey, and more extensive nutritional markers such as folate, vitamin B and D) would be needed in future studies.
